# Obituary

**Published:** 1882-09

**Authors:** 


### OBITUARY.

Prof. William H. Mussey, M.D., died in this city August 1st, of apoplexy. The medical profession in this city and vicinity has not, for a long time at least, sustained a greater loss than in the demise of Dr. Mussey.

He will be greatly missed by the public with which he was a great favorite, not only as a physician and surgeon, but in many other ways. He always had great interest in matters of public welfare. In sanitary affairs and in public education, he took an especial interest. He was engaged, professionally, in the army through the entire period of our civil war. He occupied, for part of the time at least, the highest position then attainable by the physician or surgeon. Soon after the close of the war he was appointed to the chair of surgery in the Miami Medical College, which position he held till his death. He was one of the surgeons of the Cincinnati Hospital for nearly twenty years. He has occupied many positions of importance and great responsibility, and always discharged the duties of each with marked ability and honor. "He was an earnest and sincere Christian, and carried the spirit of his Master into every department of his busy life."

His name was a household word wherever he was known personally. His reputation was national, indeed, it may with truth be said, it was world wide.

His life was an illustrious example for those who aspire to usefulness and honor.

